# Effect of body position on the redistribution of regional lung aeration during invasive and non-invasive ventilation of COVID-19 patients

**DOI:** 10.1038/s41598-022-15122-9

**Published:** 2022-06-30

**Authors:** André Dos Santos Rocha, John Diaper, Adam L. Balogh, Christophe Marti, Olivier Grosgurin, Walid Habre, Ferenc Peták, Roberta Südy

**Affiliations:** 1 Unit for Anaesthesiological Investigations, Division of Anaesthesiology, Department of Anaesthesiology, Pharmacology, Intensive Care and Emergency Medicine, University Hospitals of Geneva and University of Geneva, Rue Willy Donzé 6, 1205 Geneva, Switzerland; 2 Department of Internal Medicine, University Hospitals of Geneva, Geneva, Switzerland; 3 Department of Medical Physics and Informatics, University of Szeged, Szeged, Hungary

**Keywords:** Respiratory distress syndrome, Medical imaging

## Abstract

Severe COVID-19-related acute respiratory distress syndrome (C-ARDS) requires mechanical ventilation. While this intervention is often performed in the prone position to improve oxygenation, the underlying mechanisms responsible for the improvement in respiratory function during invasive ventilation and awake prone positioning in C-ARDS have not yet been elucidated. In this prospective observational trial, we evaluated the respiratory function of C-ARDS patients while in the supine and prone positions during invasive (n = 13) or non-invasive ventilation (n = 15). The primary endpoint was the positional change in lung regional aeration, assessed with electrical impedance tomography. Secondary endpoints included parameters of ventilation and oxygenation, volumetric capnography, respiratory system mechanics and intrapulmonary shunt fraction. In comparison to the supine position, the prone position significantly increased ventilation distribution in dorsal lung zones for patients under invasive ventilation (53.3 ± 18.3% vs. 43.8 ± 12.3%, percentage of dorsal lung aeration ± standard deviation in prone and supine positions, respectively; *p* = 0.014); whereas, regional aeration in both positions did not change during non-invasive ventilation (36.4 ± 11.4% vs. 33.7 ± 10.1%; *p* = 0.43). Prone positioning significantly improved the oxygenation both during invasive and non-invasive ventilation. For invasively ventilated patients reduced intrapulmonary shunt fraction, ventilation dead space and respiratory resistance were observed in the prone position. Oxygenation is improved during non-invasive and invasive ventilation with prone positioning in patients with C-ARDS. Different mechanisms may underly this benefit during these two ventilation modalities, driven by improved distribution of lung regional aeration, intrapulmonary shunt fraction and ventilation-perfusion matching. However, the differences in the severity of C-ARDS may have biased the sensitivity of electrical impedance tomography when comparing positional changes between the protocol groups.

Trial registration: ClinicalTrials.gov (NCT04359407) and Registered 24 April 2020, https://clinicaltrials.gov/ct2/show/NCT04359407.

## Introduction

The severe acute respiratory syndrome coronavirus 2 (SARS-CoV-2) primarily affects the respiratory system and leads to hypoxic respiratory failure, for which 5 to 49%^1^ of the patients require intensive therapy and mechanical ventilation^2,3^.

Patients with SARS-CoV-2-related acute respiratory distress syndrome (C-ARDS) present an unusual pattern of lung injury characterised by relatively well-preserved lung gas volume, but severely impaired ventilation-perfusion matching^4^. Similar to non-COVID-19-related acute respiratory distress syndrome (ARDS), atelectasis develops mainly in the dependent lung regions in C-ARDS. Furthermore, pulmonary micro-thromboembolism also contributes to the severely deteriorated lung function in C-ARDS due to pulmonary endothelial damage^5^. Thus, the vascular pathology augments the development of large dependent regions^6,7^. This gravity-related ventilation-perfusion mismatch can be potentially alleviated by placing the patient in prone position^8,9^, since this position is associated with improved oxygenation for patients with ARDS^10^ and C-ARDS^11^. Accordingly, a potential lung homogenising effect of prone positioning can be anticipated in critically ill patients with COVID-19 and thus change in body position has been suggested in the guidelines both for intubated and non-intubated patients^12^. Nevertheless, this recommendation is based mainly on meta-analysis from randomised controlled trials investigating patients with non-COVID-19-related ARDS^10^.

Despite expert consensus on promoting the prone position for patients with C-ARDS, there is a lack of firm evidence to support the beneficial effects of this therapeutic strategy in SARS-Cov-2 pneumonia, and its value has not been verified by quantitative assessment of regional ventilation distribution. In addition, there is a lack of knowledge about the mechanisms that potentially improve the lung function of mechanically ventilated C-ARDS patients in prone position. Therefore, our aim was to elucidate the differences in regional ventilation distribution between the prone and supine positions in two groups of patients with C-ARDS ventilated invasively or receiving non-invasive ventilation (NIV). We hypothesize that the prone position favours the regional redistribution of lung ventilation in comparison to the supine position, through mechanisms that might differ between invasive and non-invasive ventilation.

## Methods

### Ethical statement

After obtaining approval from the Ethics Committee of the Canton of Geneva, Switzerland (2020-00896), written informed consent to participate in the study was obtained from the patients or from their legal representatives. The study was performed in accordance with the ethical standards specified in the 1964 Declaration of Helsinki and its later amendments and reporting followed STROBE guidelines. The research protocol was registered at Clinicaltrials.gov under the number NCT04359407.

### Design and settings

In this single-centre prospective observational study we enrolled patients with C-ARDS who required respiratory support in the prone position while at the intensive or intermediate care units of the University Hospitals of Geneva between April 27th 2020 and May 10th 2021. Patients under NIV were recruited from the intermediate care unit; whereas, invasively-ventilated patients were recruited from the intensive care unit (ICU). Each patient was assessed in supine and prone positions in consecutive order depending on their position at the time of enrolment.

### Participants

**Figure 1:**
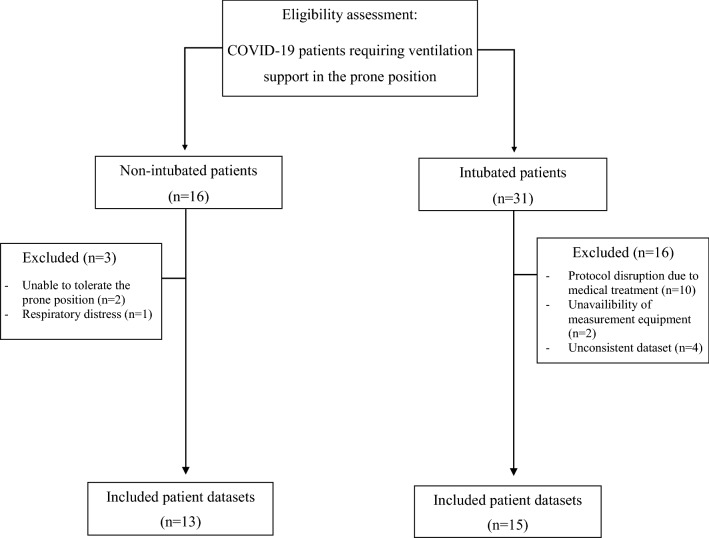
Patient flowchart.

The study flow chart is shown in Fig. 1. Patients were enrolled if (1) their respiratory condition met the Berlin definition for moderate or severe ARDS^13^, (2) they had SARS-CoV-2 infection confirmed by PCR test, (3) they were scheduled to undergo prone positioning, and (4) they were between 18 and 80 years old. Patients with pacemakers, defibrillators or other electrically active implants, with damaged skin or wound dressings that impaired skin contact of the electrodes, with chest tubes or with a history of thoracic surgery or lung resection were not included in this study.

### Endpoints

For both groups of patients receiving NIV and invasive ventilation, the primary endpoint was the relative change in regional ventilation between supine and prone positions measured with electrical impedance tomography (EIT, *see below*).

The secondary endpoints for non-invasively ventilated patients were the inspired fraction of oxygen (FiO_2_) and the peripheral oxygen saturation (SpO_2_) resulting from postural changes. Since mechanically ventilated patients were equipped with arterial and central venous catheters and mainstream volumetric capnography was performed as part of the standard procedure, the secondary endpoint in these patients were the intrapulmonary shunt fraction (Qs/Qt), ratio of arterial partial pressure of oxygen (PaO_2_) and FiO_2_, phase 3 slope of the volumetric capnogram (SIII) and ventilation dead space fractions resulting from postural change. Total respiratory system mechanics including resistance (Rrs) and compliance (Crs), blood pressure, heart rate, rhythm and vasopressor dose were also recorded.

### Data sources and measurements

#### Electrical impedance tomography

EIT was performed using the Dräger PulmoVista^®^ 500 device (Lübeck, Germany), in accordance to the international consensus statement on EIT measurements^14^. A medium, large or extra-large belt containing 16 surface electrodes was placed around the patient’s chest in the 5th intercostal space, according to the size of the thoracic cage. EIT measurements were performed in both supine and prone position for a period of five minutes, while the belt was kept in place. EIT data were generated by the injection of small electrical currents (5 mA at 50 Hz). Images of 32 × 32 pixels were reconstructed from the EIT data using the manufacturer’s algorithm. Tidal impedance variation (∆Z) from each minute were averaged in four regions of interest (ROI), defined as either quadrants or layers, and expressed as percentage of the global ∆Z (∆Z_ROI_/∆Z_global_). This variable has been demonstrated to be correlated with VT^15^ and local compliance variation^16^.

#### Respiratory data obtained during invasive and non-invasive ventilation

Non-intubated patients received ventilation support with either continuous positive airway pressure (CPAP) with a facemask (Hamilton C1, Hamilton Medical AG, Switzerland, n = 11) or with nasal high-flow therapy (Optiflow, Fisher & Paykel Healthcare, New Zealand, n = 2). Respiratory rate, FiO_2_ and CPAP pressure were registered. Intubated patients were ventilated in volume-controlled mode using a Hamilton C6 ventilator (Hamilton Medical AG, Switzerland). Respiratory parameters measured by the ventilator or set by the ICU staff to provide an adequate low-tidal ventilation were recorded, (positive end-expiratory pressure, plateau pressure [Pplat], respiratory rate, Rrs and Crs). Rrs was calculated as the ratio between the peak to plateau pressure drop and the resulting flow rate, whereas Crs is determined as the ratio between the tidal volume and the corresponding change in the difference between plateau pressure and PEEP. All patients received neuromuscular blocking agents.

#### Blood gas data

Blood gas data were collected concomitantly with EIT measurements for the intubated patients both before and after changing position. Blood gas data were not available for patients under NIV since an arterial catheter was not part of their standard of care. PaO_2_, arterial saturation of oxygen (SaO_2_) and partial pressure of carbon-dioxide (PaCO_2_), arterial and central venous haemoglobin concentrations, and central venous oxygen partial pressure and saturation (ScvO_2_) were measured. The PaO_2_/FiO_2_ ratio was calculated as the lung oxygenation index. The Qs/Qt ratio was assessed based on the modified Berggren equation^17^

$$ \frac{Qs}{{Qt}} = \frac{{CcO_{2} - CaO_{2} }}{{CcO_{2} - CvO_{2} }} $$

where CaO_2_ and CvO_2_ are the oxygen content of the arterial and central venous blood, respectively, and CcO_2_ is the capillary oxygen content.

#### Volumetric capnography

Capnographic measurements in the intubated patients were carried out by the FluxMed mainstream volumetric capnograph (MBMED, Santa Fé, Argentina). The mainstream capnography sensor (Capnostat) together with the flow sensor were inserted proximally between the Y piece and the endotracheal tube. The phase III slope of the volumetric capnogram (SIII) reflecting the emptying of alveolar gas was calculated using the FluxMed software (FluxView^®^)^18^. The ventilation dead space according to Fowler^19^ was calculated as the volume expired until the inflection point of the phase II slopes of the volumetric capnogram. The ventilation dead space fraction according to Bohr^20^ was calculated as

$$ {\text{VD}}_{{\text{B}}} /{\text{VT }} = \, ({\text{PA CO}}_{{2}} - {\text{ P}}\overline{E} {\text{CO}}_{{2}} )/{\text{PA CO}}_{{2}} $$

where PA CO_2_ is the mean alveolar partial pressure of CO_2_ estimated from the midpoint of phase III of the capnogram. PĒCO_2_ is the mixed expired partial pressure of CO_2_, determined as integrating the area under the volumetric capnogram curves and dividing the resulting values by VT.

The ventilation dead space fraction according to Enghoff^21^ was calculated as

$$ {\text{VD}}_{{\text{E}}} /{\text{VT }} = \, \left( {{\text{PaCO}}_{{2}} - {\text{ P}}\overline{E} {\text{O}}_{{2}} } \right)/{\text{PaCO}}_{{2}} $$

### Measurement biases

To avoid potential bias during the experimental protocol, the EIT belt was kept in place and the respiratory circuit was not modified between changes in body position. To avoid carryover effects, the supine or prone body position were randomly ordered based on the original position of the patients at the time of the first data collection period. After turning the patient, a 60-min stabilisation period allowed a steady state condition to be reached.

### Study sample size

Sample size calculation was based on the consideration that a 10% change in the regional ventilation is clinically relevant. This analysis revealed the need for a minimum of 13 patients to detect statistically significant differences using a paired t-test on the main outcome variable, assuming a standard deviation of a change of 10%, a power of 0.9 and alpha level of 0.05. To ensure at least 13 patients for each study group, drop-out patients were replaced by enrolling additional patients.

### Statistical methods

Numerical data are reported as mean and standard deviation. Graphical figures (boxplots) are reported using median and interquartile ranges. The normal distribution of the data was checked with the Shapiro–Wilk test. Paired t-tests were applied to assess the effect of patient position on the primary and secondary outcome variables. To test the effect of patient position in different ROI’s, two-way repeated measures of analysis of variance (ANOVA) were used with Holm–Šídák *post-hoc* analysis. The Brown-Forsythe equal variance test and the Shapiro–Wilk tests were applied prior to ANOVA. Pearson correlation test was applied to assess the relationship between the changes in PaO_2_/FiO_2_ and Qs/Qt between body positions. SigmaPlot 14 (Systat software, Inc. Chicago, IL, USA) was used for statistical analysis. A level of *p* < 0.05 was considered statistically significant, all reported p values are two-tailed.

### Ethics approval and consent to participate

The present study was approved by the Ethics Committee of the Canton of Geneva, Switzerland, under the number CCER 2020-00896. Written informed consent to participate in the study was obtained from the patients or from their legal representatives. 

## Results

### Participants

Forty-seven patients with C-ARDS requiring respiratory support in the prone position were enrolled in the study. Of the 16 patients under NIV, one patient was excluded due to respiratory distress during the observation period and two because they did not tolerate the prone position, thus 13 non-intubated patients were included in the final analysis. Data from 15 intubated patients were included in the analysis after exclusion of 16 patients due to protocol disruption for medical treatment or incomplete data acquisition for technical reasons (Fig. 1). Anthropometric and clinical characteristics of patients under non-invasive and invasive ventilation are listed in Table 1. Both groups had a majority of males, and an important prevalence of patients who were overweight and/or diagnosed with diabetes and hypertension.

**Table 1:** Anthropometric and clinical data of the patients.

	Non-intubated patients (n = 13)	Intubated patients (n = 15)
Age (years)	61 ± 9	68 ± 7
Height (cm)	171 ± 10	174 ± 5
Weight (kg)	78 ± 16	90 ± 26
Sex (female/male)	6/9	3/12
Hypertension (n)	7	12
Diabetes (n)	5	11
BMI (kg/m^2^)	26.3 ± 3.5	29.7 ± 8.3
COPD (n)	0	1
Malignancy (n)	0	2
Initial body posture (supine/prone, n)	11/2	6/9

Values are presented as mean ± standard deviation. *BMI*: Body mass index, *COPD* Chronic obstructive pulmonary disease.

### Primary endpoints

**Figure 2:**
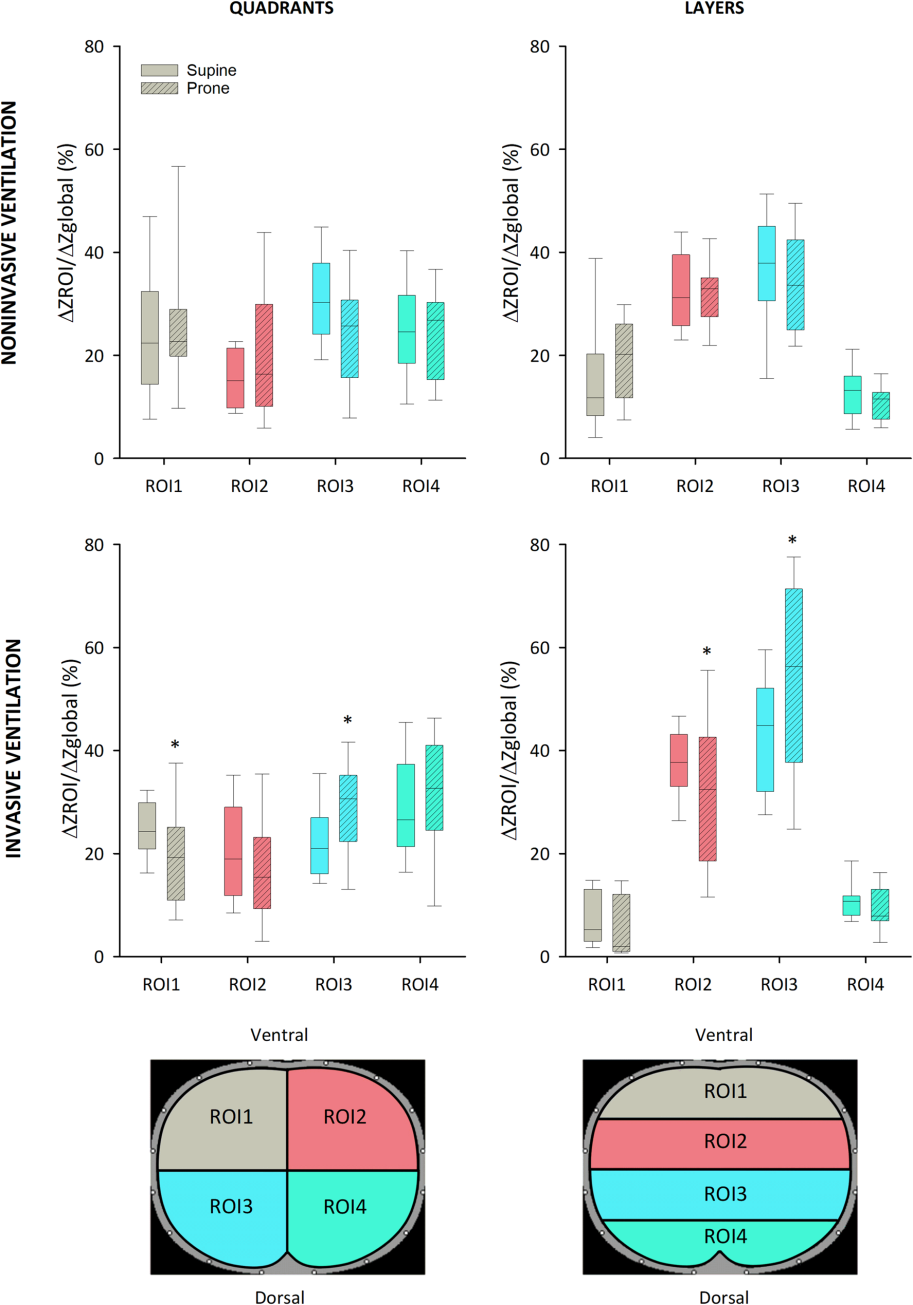
Regional ventilation assessed by regional tidal impedance variations relative to global tidal impedance variation (∆z_ROI_/∆z_global_) obtained by electrical impedance tomography for non-invasively and invasively ventilated patients. Empty boxes indicate data acquired in prone position; boxes with diagonal line pattern indicate data acquired in supine position. Regions of interest (ROIs) were defined as quadrants or layers. *: *p* < 0.05 versus supine position.

Figure 2 represents changes in ∆Z_ROI_/∆Z_global_ in the different ROIs between supine and prone position for patients requiring NIV (upper panels) and invasive mechanical ventilation (lower panels). There was no evidence for a statistical difference in the regional lung aeration between the prone and supine positions in any ROI (ventral or dorsal) for the COVID-19 patients under NIV. Conversely, for patients under invasive ventilation, prone positioning led to a significant redistribution of regional lung aeration in favour of dorsal zones (ROI 3, both in layers (53.3 ± 18.3% vs. 43.8 ± 12.3%, prone vs. supine, respectively; *p* = 0.014) and quadrants (29.3 ± 9.6% vs. 23.1 ± 7.9%; *p* = 0.012)). Further examining the regional changes in lung aeration revealed that gas redistribution occurred primarily in the left region of the lung (quadrant ROI 1 and 3).

### Secondary endpoints

**Figure 3:**
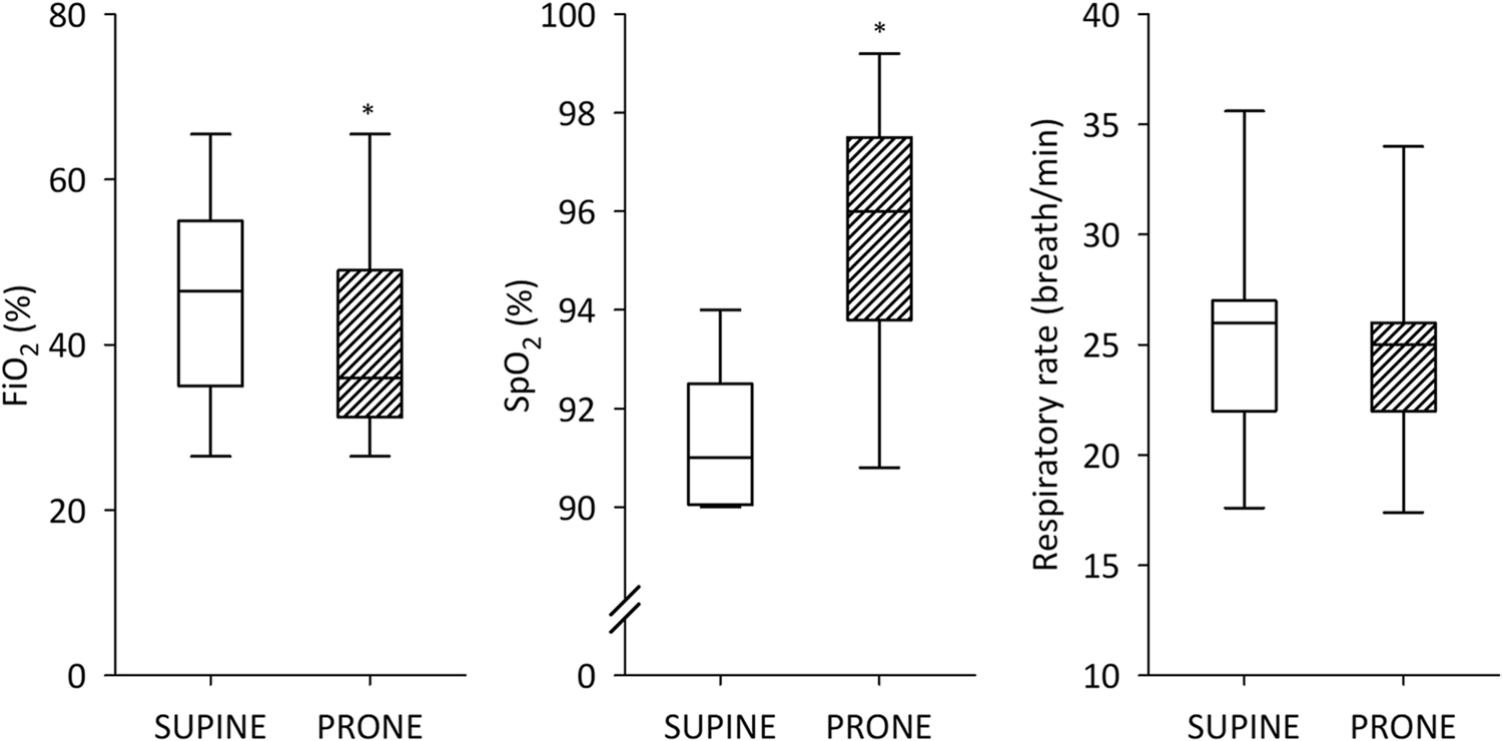
Fraction of inspired oxygen (FiO_2_), oxygen saturation (SpO_2_), and respiratory rate for noninvasively ventilated patients in supine and prone position. Empty boxes indicate data acquired from the prone position; boxes with diagonal line pattern indicate data from the supine position. *: *p* < 0.05 versus supine position.

A significant improvement in oxygenation was observed for non-invasively ventilated patients, as evidenced by increased SpO_2_ (*p* < 0.01) following the assumption of the prone position (Fig. 3). Noteworthy, the higher SpO_2_ observed in the prone position was accompanied by a statistically significant decrease in the FiO_2_. (*p* < 0.05). There was no evidence for a difference in the respiratory rate between prone and supine position under NIV (Table 2).

**Table 2:** Respiratory and haemodynamic data in prone and supine position for non-intubated and intubated patients.

		Supine position	Prone position
Non-intubated patients	CPAP (cmH_2_O)	8 ± 1	8 ± 1
	Respiratory rate (breath/min)	25.4 ± 5.5	24.7 ± 5.2
	Systolic pressure (mmHg)	127.8 ± 14.9	128.5 ± 17.9
	Diastolic pressure (mmHg)	71.0 ± 12.0	68.2 ± 9.4
	Heart rate (beats/min)	67 ± 10	69 ± 10
Intubated patients	PEEP (cmH_2_O)	10 ± 1.7	9.7 ± 1.5
	Plateau pressure (cmH_2_O)	26.9 ± 4.8	28.5 ± 4.0
	Respiratory rate (breath/min)	20.4 ± 4.2	20.7 ± 4.6
	Total respiratory resistance (cmH_2_O/l/s)	11.55 ± 0.96	10.89 ± 1.53*
	Total respiratory compliance (ml/cmH_2_O)	34.26 ± 14.27	32.96 ± 14.87
	Systolic pressure (mmHg)	117.0 ± 13.1	119.5 ± 16.2
	Diastolic pressure (mmHg)	62.5 ± 10.6	64.1 ± 9.9
	Heart rate (beats/min)	65 ± 7	66 ± 7

Values are presented as mean ± standard deviation. *CPAP* Continuous positive airway pressure, *PEEP* positive end-expiratory pressure. *: *p* < 0.05 versus supine position compared with paired t-test.

**Figure 4:**
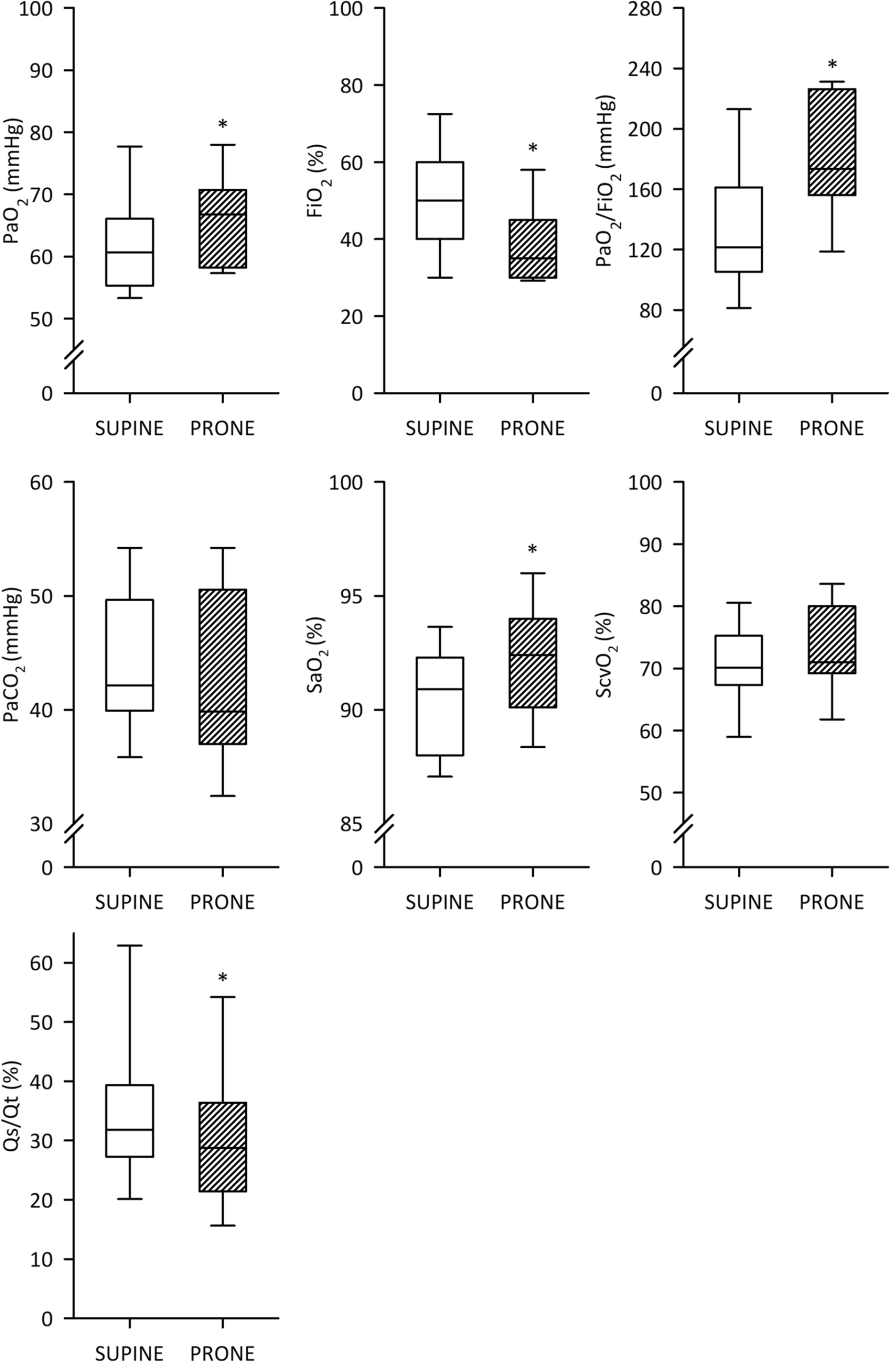
Blood gas and oxygenation data for intubated patients in supine and prone positions. Partial pressure of oxygen in the arterial blood (PaO_2_), fraction of inspired oxygen (FiO_2_), partial pressure of carbon dioxide in the arterial blood (PaO_2_), arterial oxygen saturation (SaO_2_), central venous saturation (ScvO_2_), intrapulmonary shunt fraction (Qs/Qt). Empty boxes indicate data acquired in prone position; boxes with diagonal line pattern indicate data acquired supine position. *: *p* < 0.05 versus supine position.

**Figure 5:**
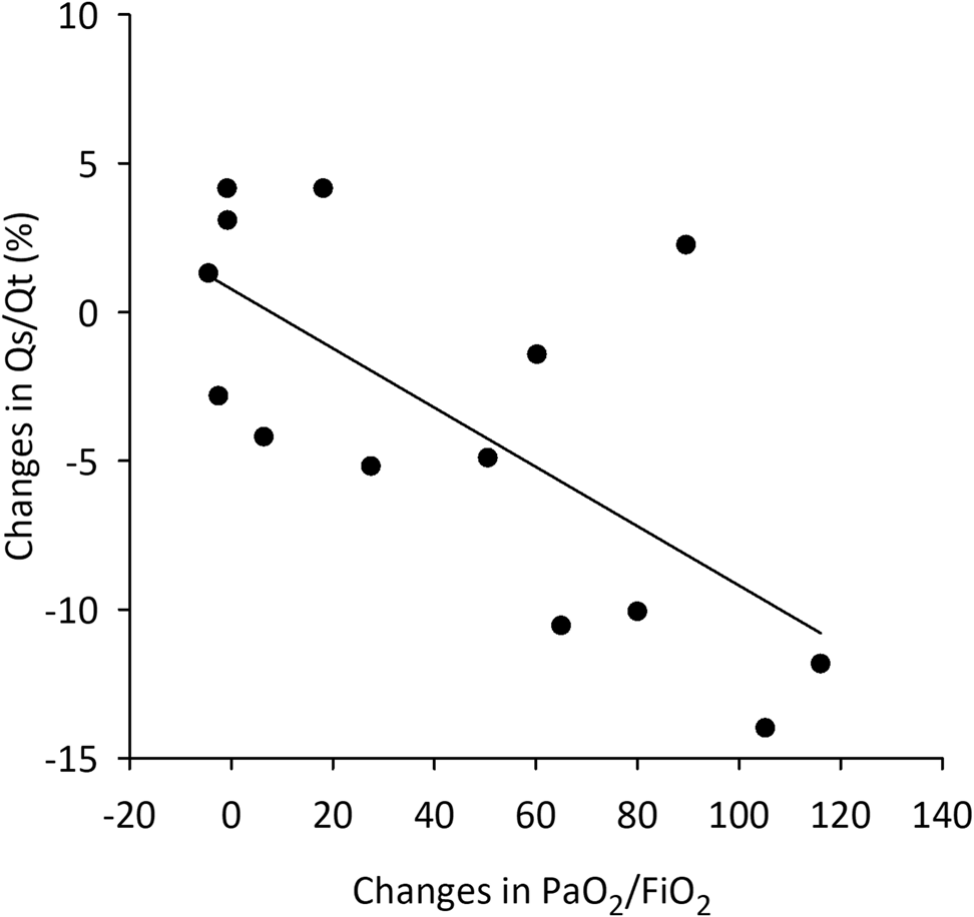
Correlation between the changes in PaO_2_/FiO_2_ and changes in Qs/Qt occurring when changing from supine to prone position. The solid line indicates the linear regression.

For patients under invasive ventilation, the gas exchange parameters are summarised in Fig. 4. A significantly higher PaO_2_ and SaO_2_ were observed along with a significantly lower FiO_2_ in the prone position (*p* < 0.05 for all), which resulted in a significantly higher PaO_2_/FiO_2_ in comparison to the supine position (*p* < 0.001). In addition, the prone position led to a significantly lower Qs/Qt in these patients (*p* < 0.05) with a negative correlation between postural changes in Qs/Qt and PaO_2_/FiO_2_ (correlation coefficient r = − 0.69, *p* < 0.01, Fig. 5). Finally, there was no evidence for a significant difference in PaCO_2_, ScvO_2_ or respiratory rate between the two positions (Fig. 4 and Table 2), as expected since the ventilator parameters were not modified between the positions.

**Figure 6:**
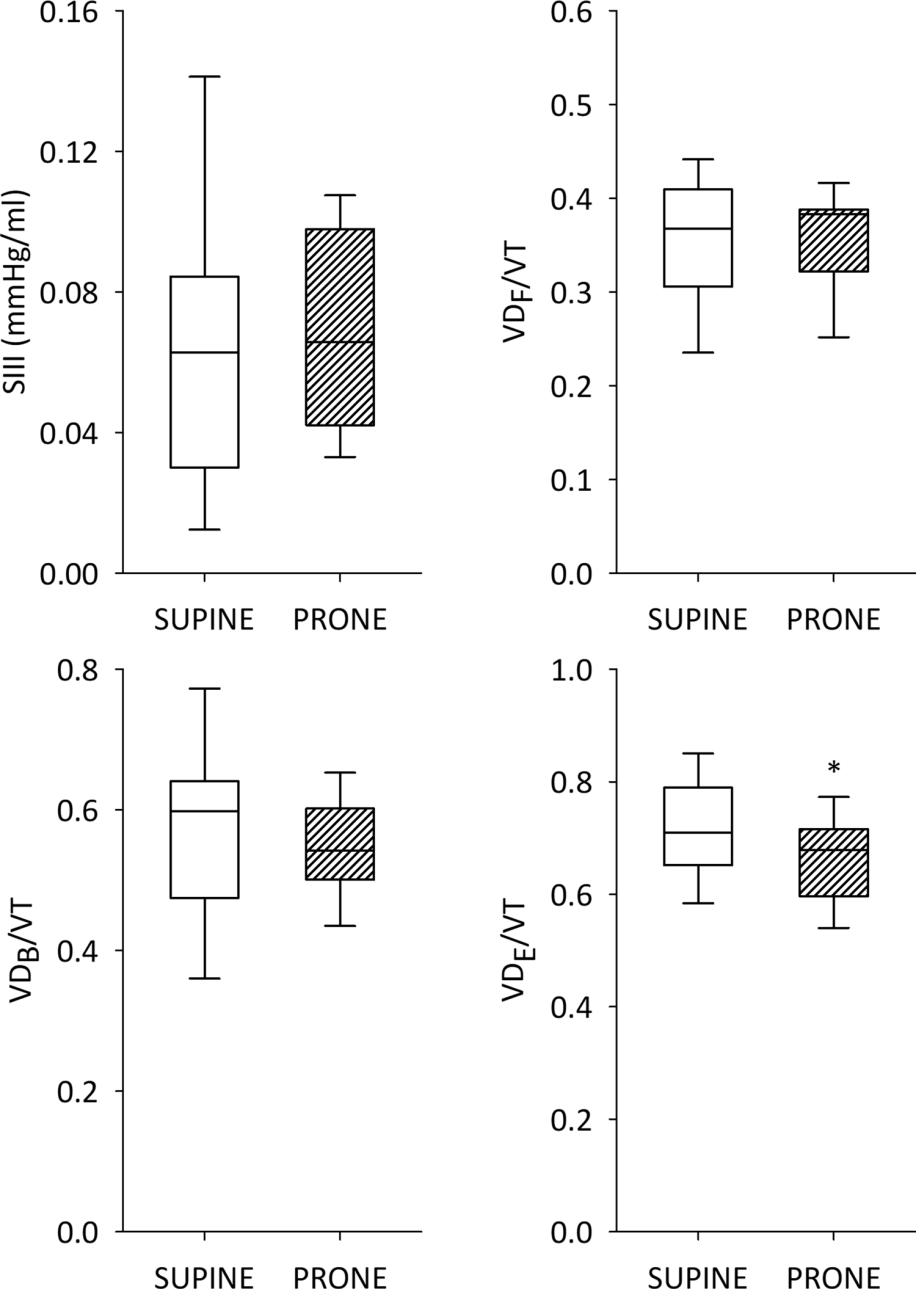
Volumetric capnography data. Volumetric phase 3 slope (SIII), ventilation dead space fractions relative to the tidal volume according to Fowler (VDf), Bohr (VDb), and Enghoff (VDe) for intubated patients in supine and prone positions. Partial pressure of oxygen in the arterial blood (PaO_2_), fraction of inspired oxygen (FiO_2_), partial pressure of carbon dioxide in the arterial blood (PaO_2_), arterial oxygen saturation (SaO_2_), central venous saturation (ScvO_2_), intrapulmonary shunt fraction (Qs/Qt). Empty boxes indicate data acquired in prone position; boxes with diagonal line pattern indicate data acquired in supine position. *: *p* < 0.05 versus supine position.

Figure 6 summarises the changes in the parameters obtained by volumetric capnography in the invasively ventilated COVID-19 patients. There was no evidence for a change in SIII or the anatomical or physiological dead space according to Bohr. However, the physiological dead space according to Enghoff decreased significantly following prone positioning (*p* < 0.02).

Respiratory and haemodynamic data are provided in Table 2. There was no evidence for a difference in any of the recorded parameters between the two positions for NIV patients. Conversely, patients under invasive ventilation demonstrated a significantly lower respiratory resistance in the prone position (*p* < 0.05).

## Discussion

In this prospective observational trial, we observed differences in the regional ventilation redistribution between invasive and NIV following changes from supine to prone position in patients with COVID-19-related ARDS. While no ventilation redistribution was evidenced in non-invasively ventilated patients, prone positioning of invasively ventilated patients led to redistribution of regional ventilation from the ventral to the dorsal lung areas. Prone positioning improved oxygenation both for patients under NIV and invasive ventilation. The improvements in ventilation and oxygenation in prone position were accompanied by a significantly lower physiological dead space, intrapulmonary shunt and respiratory resistance for intubated COVID-19 patients.

We studied two different populations with moderate-to-severe C-ARDS, requiring either NIV or invasive ventilation for acute respiratory failure. The clinical and demographic characteristics of our study subjects correspond to those previously reported in the literature, with a majority of male patients, over 60 years of age, exhibiting an important prevalence of overweight, diabetes and hypertension^22^. The regional ventilation distribution was assessed in the present study with EIT, a well-validated dynamic, real-time imaging technique^23,24^, which has been used to characterise or to guide the ventilation strategy in COVID-19 patients^25–27^. The dorsal redistribution of regional ventilation observed only for invasively ventilated patients with C-ARDS may be attributed in part to recruitment of atelectatic areas, which are more pronounced in invasively ventilated patients than those under awake prone positioning. Interestingly, lateral differences between right and left lungs were observed in the redistribution of ventilation (Fig. 2) with prone positioning. This can be explained by the findings that COVID-19 related pneumonia deteriorates more severely the right lung^28,29^. Thus, the right lung may have less ability for improvement in the time-window studied in the present protocol. Additionally, the improvement in regional ventilation observed for the intubated COVID-19 patients was reflected in a significant decrease in Qs/Qt and Enghoff dead space, which further indicates an improvement in ventilation-perfusion matching with subsequent improvement in oxygenation indices. Of note, there was a strong correlation between the postural changes in Qs/Qt and in PaO_2_/FiO_2_ (Fig. 5), demonstrating that the decrease in intrapulmonary shunt is responsible for the improved oxygenation. However, despite the improvement in Qs/Qt, the ventilation-perfusion mismatch was still considerable in the prone position.

A hallmark finding of this study is that oxygenation was improved both under invasive and non-invasive ventilation and was associated even with a simultaneous reduction in FiO_2_. Since the central feature of SARS-Cov-2 pneumonia is hypoxaemic respiratory failure^1^, supplemental oxygen is a cornerstone in all therapy guidelines. However, excessive oxygen has deleterious effects on lung function, including toxicity through oxygen free radicals and oxidative stress, leading to increased alveolo-capillary permeability, decreased alveolar macrophage viability and lung fibrosis^30–34^. Therefore, the lower FiO_2_ achieved in the prone position is of paramount importance. In contrast to previous reports on the reduction of the respiratory rate (RR) during awake prone positioning^35,36^, we observed no change in RR in our cohort of patients under NIV despite an improvement in oxygenation.

Since regional aeration was not significantly modified by prone positioning in NIV patients, we can assume that the benefit in oxygenation is predominantly explained by the redistribution of pulmonary blood flow and optimisation of ventilation-perfusion matching, rather than alveolar recruitment or aeration change as speculated by previous literature^37^.

The data on volumetric capnography provides additional insight into the respiratory effects of C-ARDS and body position. The phase 3 slope is not significantly modified by body position and presents values that are normal or even lower than normal^38^. While a markedly decreased phase 3 slope was reported earlier for patients with ARDS^18^, our findings suggest that C-ARDS differs from non-COVID ARDS in this aspect. This finding suggests that, in C-ARDS, regional ventilation and perfusion of the open lung regions might be more homogeneous than in non-COVID ARDS. Conversely, in terms of Bohr and Enghoff dead space, the changes are similar to those observed in non-COVID ARDS^39^ and were markedly higher than those obtained for ventilated patients with healthy lungs^38,40^. A marked elevation in the difference between the Bohr and Fowler dead spaces (VD_B_–VD_F_) is observed regardless of the body position, which suggests the presence of large lung regions that are ventilated but not perfused. Similarly, the difference between the Enghoff and Bohr dead spaces (VD_E_–VD_B_) is much higher than in healthy ventilated patients^38,40^, indicating the development of alveolar regions with intrapulmonary shunt. Moreover, prone positioning significantly decreased the Enghoff’s dead space VD_E_ for intubated patients, suggesting a beneficial redistribution of lung aeration. The Enghoff’s dead space VD_E_ incorporates the intrapulmonary shunt in addition to the VD_B_^41^. Altogether, these findings demonstrate that simultaneous respiratory and circulatory changes are responsible for the ventilation–perfusion mismatch observed for patients with C-ARDS.

Some limitations of this study warrant consideration. First, the measurements with EIT were solely presented as the percentage of aeration instead of as absolute impedance or volume units; this data presentation reduces the bias related to technical issues related to intra- and inter-subject impedance variability. Second, the respiratory circuit used in this study (instrumental dead space) comprised the endotracheal tube, the closed-circuit suction, the flowmeter, the mainstream capnograph and a bacterial filter with continuous humidification; this circuit dead space was similar in all patients and corresponds to the standard circuit in routine clinical practice. This instrumental dead space may have contributed to the VD_F_ without affecting changes in the other outcomes. Third, the heterogeneous nature of both the lung injury and the disease severity between patients led to considerable variances in the estimated endpoints. Thus, the precise benefits of prone position for each individual may vary over time, depending on the rapidly changing pathological aspects of the disease^42,43^. Nevertheless, despite the aforementioned variability, we demonstrated a consistent improvement in oxygenation that is in accordance with previous reports^35–37,44^. Fourth, despite an estimated sample size of 13 patients per group, we had to enrol 31 intubated patients to have complete datasets for both prone and supine positions, due to the fact that these ICU patients often required medical care interventions that disrupted the research protocol procedures. Finally, this study reports a single-centre trial; however, the magnitude of the improvement in oxygenation and lung function along with the clinical characteristics of the study subjects likely permit a confident generalisability of our findings to the population of patients with moderate-to-severe C-ARDS requiring ventilatory support in the prone position.

## Conclusions

In summary, our results provide additional evidence for the benefit of prone positioning for patients with moderate-to-severe C-ARDS. The benefits under NIV and invasive ventilation are governed by different underlying mechanisms. For invasively ventilated patients the improvement in oxygenation is driven by the redistribution of lung aeration and optimised ventilation-perfusion matching, leading to a decrease in physiological dead space and in intrapulmonary shunt fraction. During awake prone positioning, the lack of change in the distribution of aeration suggests that other mechanisms are responsible for the oxygenation benefits. Further studies are warranted to evaluate the factors contributing to the improved gas exchange during awake prone positioning.

## Data Availability

The datasets used and/or analysed during the current study are available from the corresponding author on reasonable request.

## Abbreviations

ARDS: Acute respiratory distress syndrome
C-ARDS: COVID-19-related acute respiratory distress syndrome
CPAP: Continuous positive airway pressure
Crs: Total respiratory system compliance
EIT: Electrical impedance tomography
FiO_2_: Inspired fraction of oxygen
ICU: Intensive care unit
NIV: Non-invasive ventilation
PaO_2_: Arterial partial pressure of oxygen
PaCO_2_: Partial pressure of carbon-dioxide
Pplat: Plateau pressure
Qs/Qt: Intrapulmonary shunt fraction
ROI: Regions of interest
Rrs: Total respiratory system resistance
SaO_2_: Arterial saturation of oxygen
SARS-CoV-2: Severe acute respiratory syndrome coronavirus 2
ScvO_2_: Central venous oxygen partial saturation
SIII: Phase 3 slope of the volumetric capnogram
SpO_2_: Oxygen saturation
VD_B_: Ventilation dead space fraction according to Bohr
VD_E_: Ventilation dead space fraction according to Enghoff
VD_F_: Ventilation dead space fraction according to Fowler
VT: Tidal volume
